# Exenatide Attenuates Doxorubicin-Induced Acute Hepatic Injury Through Modulation of SIRT1-HMGB1 Signaling and Oxidative Stress

**DOI:** 10.3390/ph19071086

**Published:** 2026-07-15

**Authors:** Haluk Kerim Karakullukcu, Serdar Savaş Gül, Hatice Aygun, Murat Kalın, Mina Karakullukcu, Aylin Arslan, Ömer Faruk Özkan, Gülçin Ercan

**Affiliations:** 1 Department of General Surgery, Sultan 2. Abdulhamid Han Educational and Research Hospital, University of Health Sciences, Istanbul 34668, Turkey; halukkarakullukcu@gmail.com (H.K.K.); murat.kalin@hotmail.com (M.K.); omerfaruk.ozkan@sbu.edu.tr (Ö.F.Ö.); ghepgul@hotmail.com (G.E.); 2 Department of Nuclear Medicine, Faculty of Medicine, Lokman Hekim University, Ankara 06530, Turkey; serdar.gul@lokmanhekim.edu.tr; 3 Neuroscience Laboratory, BAMER, Biruni University, Istanbul 34010, Turkey; 4 Department of Oncology, Sultan 2. Abdulhamid Han Educational and Research Hospital, University of Health Sciences, Istanbul 34668, Turkey; mina.zoralioglu@gmail.com; 5 Department of General Surgery, Istanbul Prof. Dr. Cemil Taşçıoğlu City Hospital, Health Sciences University, Istanbul 34668, Turkey; aylinarslan53@outlook.com

**Keywords:** doxorubicin, exenatide, hepatotoxicity, oxidative stress, inflammation, SIRT1, HMGB1, NF-κB, ^99m^Tc-pyrophosphate, scintigraphy

## Abstract

**Background:** Doxorubicin is an effective antineoplastic agent, but its use is constrained by off-target toxicities, including acute liver injury driven by mitochondrial dysfunction, oxidative stress, sterile inflammation, and redox-sensitive signaling pathways. Exenatide, a glucagon-like peptide-1 receptor agonist, exerts antioxidant and anti-inflammatory effects in several experimental liver injury models, but its role in doxorubicin-induced hepatic injury has not been investigated to our knowledge. This study evaluated whether exenatide attenuates acute doxorubicin-induced hepatic injury and examined whether the observed biochemical changes are consistent with modulation of oxidative stress and SIRT1–HMGB1/NF-κB-related signaling. **Methods:** Male Wistar albino rats were allocated to four groups (n = 7/group): control, exenatide, doxorubicin, and exenatide + doxorubicin. Exenatide (10 µg/kg/day, intraperitoneally) was administered for seven days. Doxorubicin was given intraperitoneally on days 5–7 at a cumulative dose of 18 mg/kg. The pretreatment design was used to test preventive attenuation rather than reversal of established injury. Hepatic injury-associated enzyme activities, serum HMGB1, hepatic SIRT1, NF-κB, TNF-α, IL-6, IL-10, malondialdehyde, glutathione, total antioxidant status, total oxidant status, nitric oxide, and hepatic ^99m^Tc-pyrophosphate uptake were assessed. **Results:** Doxorubicin substantially increased intrahepatic ALT and AST activities, serum HMGB1, hepatic NF-κB, TNF-α, IL-6, malondialdehyde, total oxidant status, nitric oxide, and hepatic ^99m^Tc-pyrophosphate uptake, while reducing SIRT1, IL-10, glutathione, and total antioxidant status. Relative to the doxorubicin group, exenatide lowered HMGB1 by 48.3%, hepatic ^99m^Tc-pyrophosphate uptake by 48.7%, malondialdehyde by 45.0%, total oxidant status by 37.3%, nitric oxide by 40.7%, NF-κB by 40.4%, TNF-α by 48.6%, and IL-6 by 41.1%, while increasing SIRT1 by 91.7%, IL-10 by 115%, glutathione by 115%, and total antioxidant status by 87.8%. None of the altered parameters returned completely to control levels, indicating attenuation rather than full normalization of acute injury-related changes. **Conclusions:** Exenatide attenuated doxorubicin-induced acute hepatic injury in this rat model and was associated with reduced oxidative stress, reduced inflammatory activation, higher hepatic SIRT1 levels, lower serum HMGB1 levels, and reduced hepatic ^99m^Tc-pyrophosphate uptake. The findings are consistent with involvement of SIRT1–HMGB1/NF-κB-related signaling, but they do not establish causality. Hepatic ^99m^Tc-pyrophosphate uptake should be interpreted as an exploratory imaging correlate of tissue injury rather than an established liver biomarker. Additional studies incorporating histopathology, immunohistochemistry, functional liver indices, mechanistic validation of SIRT1, and tumor-bearing models are required before translational conclusions can be drawn.

## 1. Introduction

Doxorubicin remains a cornerstone of systemic therapy for many solid and hematologic malignancies, but its clinical utility is constrained by cumulative toxicity in non-tumor tissues, including the liver, which is central to drug uptake, biotransformation, and detoxification [1,2].

Experimental and clinical evidence indicate that doxorubicin-induced hepatotoxicity involves excessive generation of reactive oxygen species, mitochondrial dysfunction, lipid peroxidation in membranes, inflammatory amplification, and hepatocyte death, thereby producing an acute biochemical profile consistent with toxic liver injury [3,4,5,6]. A growing body of evidence suggests that the interaction among oxidative stress, SIRT1, HMGB1, and NF-κB is biologically relevant in toxic liver injury [7,8,9].

SIRT1 is an NAD+-dependent deacetylase that contributes to mitochondrial homeostasis, antioxidant defense, inflammatory regulation, autophagy, and cell survival in hepatic tissue under stress conditions [8,10,11]. HMGB1, in contrast, is a damage-associated molecular pattern released by stressed or dying cells that can propagate sterile inflammation through pattern-recognition pathways, including TLR4- and RAGE-linked signaling, with downstream activation of NF-κB-dependent transcriptional programs [7,9]. NF-κB activation promotes the expression of TNF-α, IL-6, inducible nitric oxide synthase, and related mediators that may further intensify tissue injury, whereas SIRT1 has been linked to the suppression of inflammatory signaling and the regulation of HMGB1-related responses in several injury contexts [4,9]. Accordingly, the SIRT1–HMGB1–NF-κB axis provides a plausible mechanistic framework for interpreting doxorubicin-induced hepatic injury, although direct causality requires dedicated interventional validation rather than inference from biomarker shifts alone [8,9].

Exenatide is a glucagon-like peptide-1 receptor agonist widely used in type 2 diabetes and has demonstrated antioxidant, anti-inflammatory, and hepatometabolic benefits in experimental models of liver disease [12,13]. Prior studies have reported that exenatide or exendin-4 can reduce steatosis, attenuate oxidant burden, improve transaminase profiles, and suppress inflammatory signaling in models of non-alcoholic fatty liver disease, non-alcoholic steatohepatitis, steatotic ischemia–reperfusion injury, and experimental hepatic steatosis [13,14,15]. These effects have been associated with improved mitochondrial function, modulation of redox-sensitive pathways, and dampening of inflammatory cascades, including mechanisms linked to SIRT1 signaling and HMGB1-related inflammatory control [15,16]. To the best of our knowledge, no previous experimental study has specifically investigated exenatide in acute doxorubicin-induced hepatic injury while simultaneously evaluating oxidative stress, inflammatory mediators, the SIRT1-HMGB1-NF-κB signaling axis, and hepatic ^99m^Tc-pyrophosphate imaging.

An additional novel aspect of the present study is the evaluation of hepatic ^99m^Tc-pyrophosphate uptake as an exploratory imaging correlate of acute doxorubicin-induced liver injury [17,18,19]. ^99m^Tc-pyrophosphate is classically recognized as a necrosis-avid tracer in myocardial injury, whereas historical nuclear medicine literature has also described hepatic uptake in severe hepatocellular necrosis [17,19]. Because these hepatic observations are limited and predate contemporary molecular liver imaging, hepatic ^99m^Tc-pyrophosphate uptake should presently be regarded as an exploratory imaging correlate of tissue injury rather than an established liver biomarker.

Accordingly, the present study aimed to determine whether exenatide attenuates acute doxorubicin-induced hepatic injury in rats and whether the overall pattern of biochemical and scintigraphic changes is consistent with reduced oxidative stress, reduced inflammatory activation, and modulation of SIRT1-related homeostatic signaling.

## 2. Results

### 2.1. Hepatic ALT and AST

**Figure 1:**
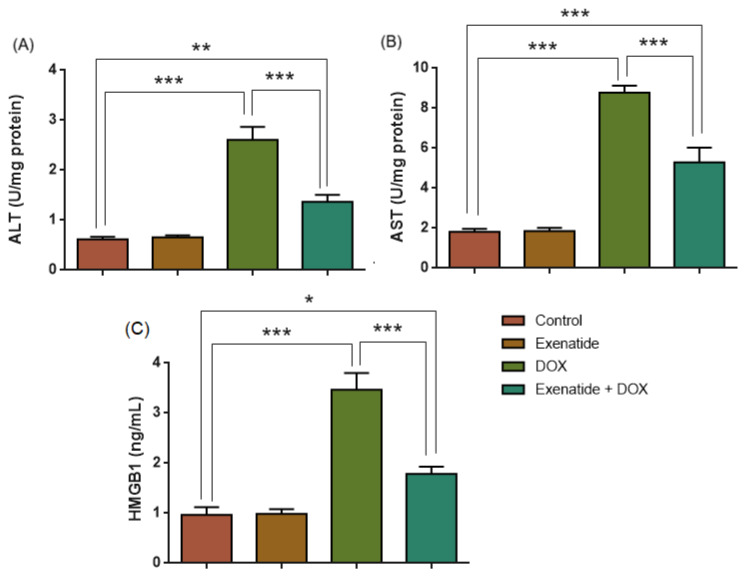
Effects of exenatide on injury-associated biochemical markers in doxorubicin-induced acute hepatic injury. (**A**) Intrahepatic ALT activity, (**B**) intrahepatic AST activity, and (**C**) serum HMGB1 levels. Doxorubicin increased all three measures, whereas exenatide significantly attenuated these changes without fully restoring control values. Data are presented as mean ± SEM (n = 7/group). Statistical comparisons were performed using one-way ANOVA followed by Tukey’s post hoc test (* *p* < 0.05, ** *p* < 0.01, *** *p* < 0.001).

As shown in Table 1 and Figure 1, intrahepatic ALT and AST activities differed significantly among groups (ALT: F(3,24) = 37.99, *p* < 0.0001; AST: F(3,24) = 61.87, *p* < 0.0001). Compared with the control group, the DOX group exhibited significantly higher intrahepatic ALT activity (2.61 ± 0.26 vs. 0.62 ± 0.04 U/mg protein; *p* < 0.0001), corresponding to an approximately 4.2-fold (321%) increase, and significantly higher AST activity (8.78 ± 0.34 vs. 1.82 ± 0.15 U/mg protein; *p* < 0.0001), corresponding to an approximately 4.8-fold (382%) increase. Compared with the DOX group, exenatide reduced intrahepatic ALT activity to 1.37 ± 0.14 U/mg protein and AST activity to 5.28 ± 0.74 U/mg protein (both *p* < 0.0001). Compared with the doxorubicin group, exenatide reduced ALT by 47.5% and AST by 39.9%, indicating a biologically meaningful attenuation of injury-associated biochemical alterations. Exenatide administration alone did not significantly alter hepatic ALT or AST activities compared with the control group (ALT: 0.66 ± 0.04 vs. 0.62 ± 0.04 U/mg protein, *p* = 0.9980; AST: 1.86 ± 0.16 vs. 1.82 ± 0.15 U/mg protein, *p* = 0.9999).

### 2.2. HMGB1 Levels

As illustrated in Figure 1 and Table 1, serum HMGB1 levels differed significantly among the experimental groups (F(3,24) = 32.62, *p* < 0.0001). Compared with the control group, the DOX group exhibited significantly higher serum HMGB1 levels (3.46 ± 0.34 vs. 0.96 ± 0.16; *p* < 0.0001), corresponding to an approximately 3.6-fold (260%) increase. Compared with the DOX group, exenatide treatment reduced serum HMGB1 levels to 1.79 ± 0.14 (*p* = 0.042), corresponding to a 48.3% reduction. Although HMGB1 levels remained above those of the control group, these findings indicate substantial attenuation of the injury-associated inflammatory response. Exenatide administration alone did not significantly alter serum HMGB1 levels compared with the control group (0.99 ± 0.09 vs. 0.96 ± 0.16; *p* > 0.05).

### 2.3. Scintigraphic Evaluation of Hepatic Injury

**Figure 2:**
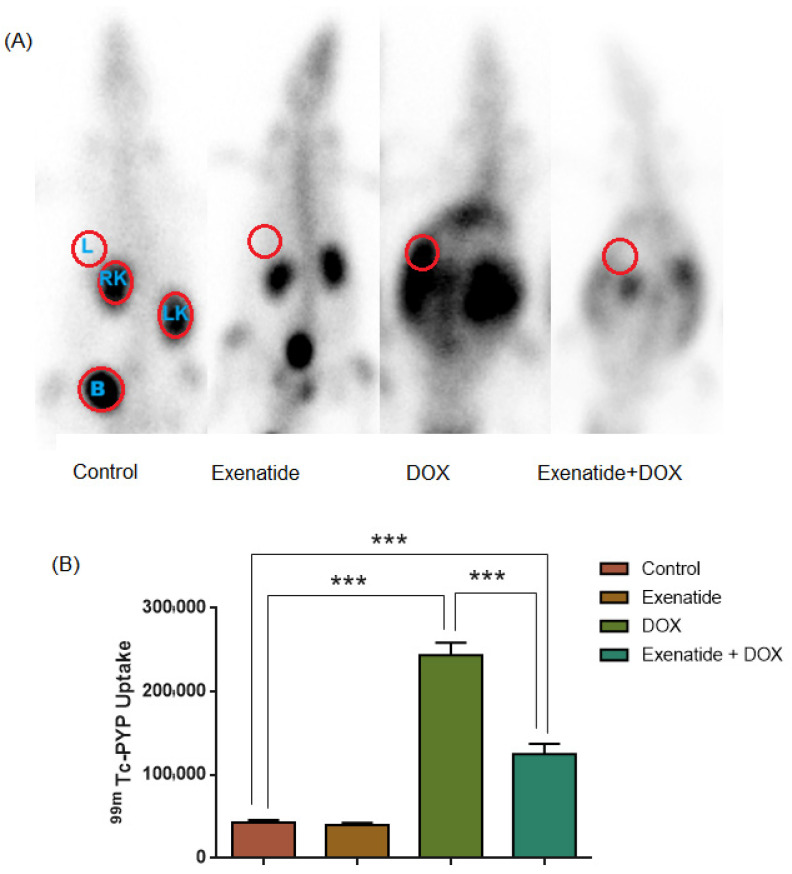
Scintigraphic evaluation of hepatic ^99m^Tc-pyrophosphate (^99m^Tc-PYP) uptake. (**A**) Representative static scintigraphic images from each experimental group showing the manually placed circular region of interest (ROI) over the anatomical projection of the liver used for quantitative analysis. L, liver; RK, right kidney; LK, left kidney; B, urinary bladder. (**B**) Quantitative analysis of hepatic ^99m^Tc-PYP uptake. Doxorubicin increased hepatic radiotracer uptake, whereas exenatide significantly attenuated this increase in the Exenatide + DOX group. Data are presented as mean ± SEM (n = 7 per group). Statistical comparisons were performed using one-way ANOVA followed by Tukey’s post hoc test. *** *p* < 0.001.

As illustrated in Figure 2 and Table 1, hepatic ^99m^Tc-pyrophosphate (^99m^Tc-PYP) uptake differed significantly among the experimental groups (F(3,24) = 93.50, *p* < 0.0001). Compared with the control group, the DOX group exhibited significantly higher hepatic scintigraphic uptake (243,609 ± 14,897 vs. 42,874 ± 2956; *p* < 0.0001), corresponding to an approximately 5.68-fold (468%) increase. Compared with the DOX group, exenatide treatment reduced hepatic scintigraphic uptake to 125,081 ± 12,404 (*p* < 0.0001), corresponding to a 48.7% reduction. Although hepatic ^99m^Tc-PYP uptake remained higher than that of the control group, these findings indicate substantial attenuation of injury-associated radiotracer accumulation. Exenatide administration alone did not significantly alter hepatic scintigraphic uptake compared with the control group (39,929 ± 2657 vs. 42,874 ± 2956; *p* > 0.05).

### 2.4. SIRT1 Levels

**Figure 3:**
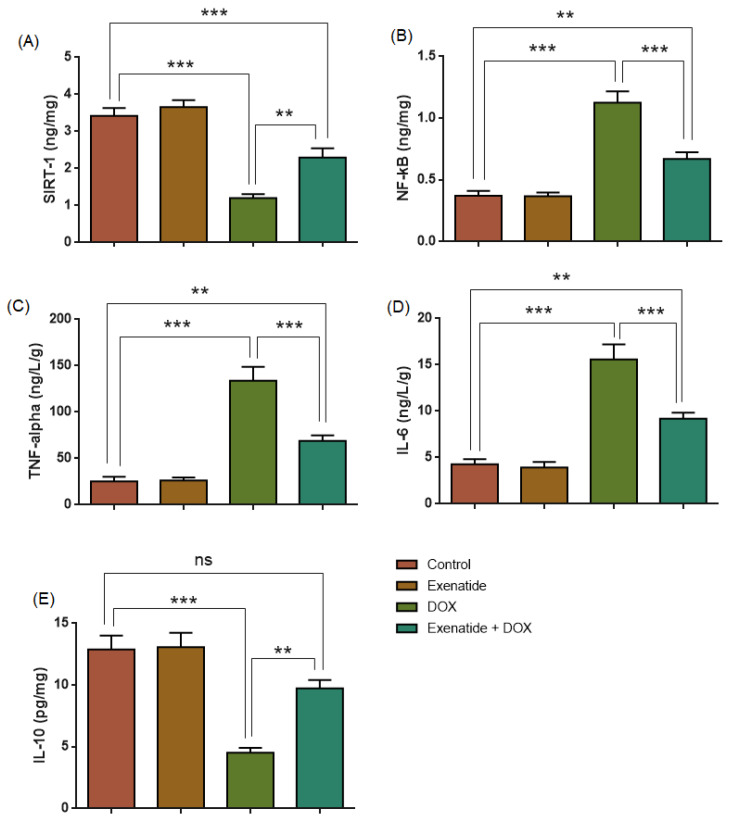
Effects of exenatide on hepatic SIRT1 and inflammatory mediators in doxorubicin-induced acute hepatic injury. (**A**) Hepatic SIRT1, (**B**) hepatic NF-κB, (**C**) hepatic TNF-α, (**D**) hepatic IL-6, and (**E**) hepatic IL-10 levels. Doxorubicin decreased SIRT1 and IL-10 while increasing NF-κB, TNF-α, and IL-6. Exenatide significantly attenuated these alterations without fully restoring the inflammatory profile to control levels. Data are presented as mean ± SEM (n = 7/group). Statistical comparisons were performed using one-way ANOVA followed by Tukey’s post hoc test (** *p* < 0.01, *** *p* < 0.001); ns, not significant.

As shown in Figure 3 and Table 2, hepatic SIRT1 levels differed significantly among the experimental groups (F(3,24) = 32.53, *p* < 0.0001). Compared with the control group, the DOX group exhibited significantly lower hepatic SIRT1 levels (1.20 ± 0.11 vs. 3.42 ± 0.21; *p* < 0.0001), corresponding to an approximately 65% decrease. Compared with the DOX group, exenatide increased hepatic SIRT1 levels to 2.30 ± 0.25 (*p* = 0.0035), corresponding to a 91.7% increase. Exenatide administration alone did not significantly alter SIRT1 levels compared with the control group (3.66 ± 0.19 vs. 3.42 ± 0.21; *p* > 0.05).

### 2.5. Inflammatory and Anti-Inflammatory Biomarkers

As demonstrated in Figure 3 and Table 2, inflammatory biomarkers differed significantly among the experimental groups. One-way ANOVA revealed significant differences for NF-κB (F(3,24) = 37.03, *p* < 0.0001), TNF-α (F(3,24) = 34.72, *p* < 0.0001), and IL-6 (F(3,24) = 31.63, *p* < 0.0001). The DOX group exhibited higher levels of NF-κB, TNF-α, and IL-6 compared with the control group (NF-κB: 1.125 ± 0.093 vs. 0.372 ± 0.038; TNF-α: 133.73 ± 15.02 vs. 25.44 ± 4.85; IL-6: 15.58 ± 1.63 vs. 4.29 ± 0.54, all *p* < 0.0001). Compared with the control group, the DOX group exhibited approximately 3.02-fold (202%), 5.26-fold (426%), and 3.63-fold (263%) increases in NF-κB, TNF-α, and IL-6 levels, respectively, reflecting a marked inflammatory response following doxorubicin administration.

In contrast, the anti-inflammatory cytokine IL-10 exhibited the opposite pattern. Compared with the control group, the DOX group showed significantly lower IL-10 levels (4.53 ± 0.39 vs. 12.90 ± 1.12; *p* < 0.0001), corresponding to an approximately 65% (2.85-fold) decrease.

Exenatide treatment attenuated these inflammatory alterations. In the Exenatide + DOX group, NF-κB, TNF-α, and IL-6 levels decreased to 0.671 ± 0.053, 68.73 ± 6.26, and 9.17 ± 0.66, corresponding to reductions of 40.4%, 48.6%, and 41.1%, respectively, compared with the DOX group. In parallel, IL-10 levels increased to 9.74 ± 0.67, representing an approximate 115% increase compared with the DOX group, although values remained slightly lower than those observed in the control group. Exenatide administration alone did not significantly alter NF-κB, TNF-α, IL-6, or IL-10 levels compared with the control group (all *p* > 0.05).

### 2.6. Oxidative Stress Biomarkers

**Figure 4:**
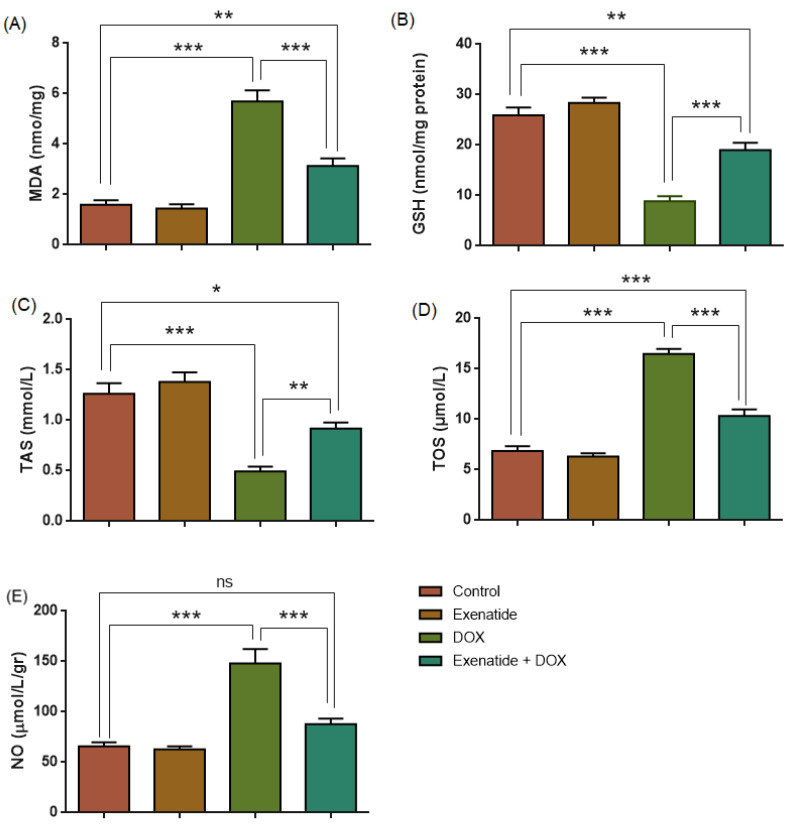
Effects of exenatide on hepatic oxidative and nitrosative stress markers in doxorubicin-induced acute hepatic injury. (**A**) Malondialdehyde (MDA), (**B**) reduced glutathione (GSH), (**C**) total antioxidant status (TAS), (**D**) total oxidant status (TOS), and (**E**) nitric oxide (NO). Doxorubicin increased oxidant and nitrosative stress markers while decreasing antioxidant defense markers. Exenatide significantly attenuated these alterations without fully restoring control values. Data are presented as mean ± SEM (n = 7/group). Statistical comparisons were performed using one-way ANOVA followed by Tukey’s post hoc test (* *p* < 0.05, ** *p* < 0.01, *** *p* < 0.001); ns, not significant.

As illustrated in Figure 4 and Table 3, oxidative stress biomarkers differed significantly among the experimental groups. One-way ANOVA revealed significant differences for MDA (F(3,24) = 43.61, *p* < 0.0001), GSH (F(3,24) = 45.16, *p* < 0.0001), TAS (F(3,24) = 24.83, *p* < 0.0001), and TOS (F(3,24) = 84.18, *p* < 0.0001).

The DOX group increased oxidative stress compared with the control group, as reflected by elevated MDA and TOS levels and reduced GSH and TAS levels (MDA: 5.69 ± 0.45 vs. 1.59 ± 0.19; TOS: 16.48 ± 0.52 vs. 6.86 ± 0.49; GSH: 8.85 ± 1.03 vs. 25.93 ± 1.56; TAS: 0.49 ± 0.05 vs. 1.26 ± 0.10, all *p* < 0.0001). When the mean control values were taken as the baseline, the DOX group showed a 3.58-fold increase in MDA and a 2.40-fold increase in TOS, accompanied by 65.9% and 61.1% decreases in GSH and TAS, respectively, indicating pronounced oxidative stress following doxorubicin administration.

Compared with the DOX group, exenatide significantly reduced MDA and TOS levels to 3.13 ± 0.30 and 10.34 ± 0.66, corresponding to reductions of 45.0% and 37.3%, respectively (both *p* < 0.0001). In parallel, GSH and TAS levels increased to 19.03 ± 1.47 and 0.92 ± 0.06, corresponding to increases of 115% and 87.8%, respectively (both *p* < 0.0001). Exenatide administration alone did not significantly alter MDA, TOS, GSH, or TAS levels compared with the control group (all *p* > 0.05). Collectively, these findings indicate that exenatide attenuated doxorubicin-associated alterations in oxidative stress markers.

### 2.7. Nitric Oxide (NO) Levels

As illustrated in Figure 4 and Table 3, nitric oxide (NO) levels differed significantly among the experimental groups (F(3,24) = 23.64, *p* < 0.0001). Compared with the control group, the DOX group exhibited significantly higher NO levels (148.01 ± 14.47 vs. 65.88 ± 3.99; *p* < 0.0001), corresponding to an approximately 2.25-fold (125%) increase. Compared with the DOX group, exenatide treatment reduced NO levels to 87.80 ± 5.44 (*p* = 0.0001), corresponding to a 40.7% reduction. Exenatide administration alone did not significantly alter NO levels compared with the control group (62.81 ± 3.07 vs. 65.88 ± 3.99; *p* > 0.05). Collectively, these findings indicate that exenatide attenuated doxorubicin-associated alterations in nitric oxide levels.

### 2.8. Correlation Analysis

**Figure 5:**
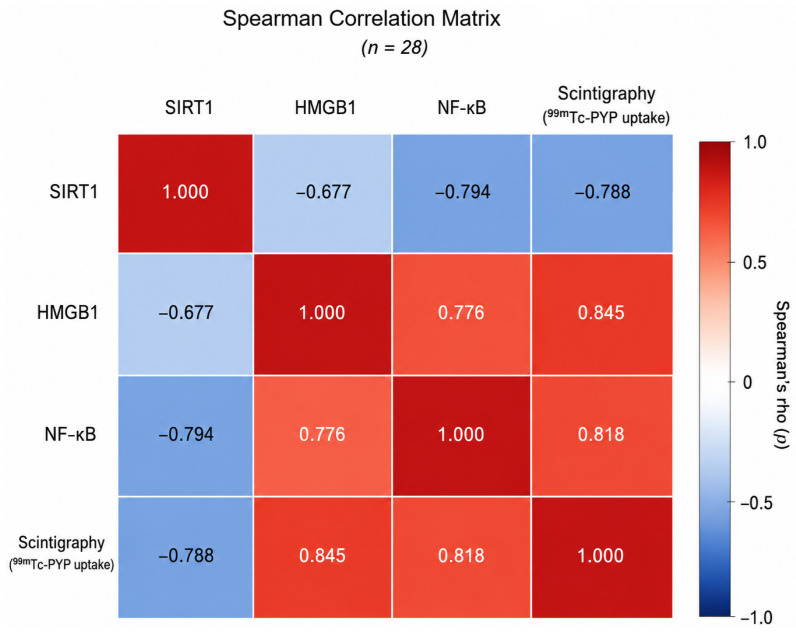
Spearman correlation matrix demonstrating the relationships among hepatic SIRT1, serum HMGB1, hepatic NF-κB, and hepatic ^99m^Tc-pyrophosphate (^99m^Tc-PYP) uptake in all experimental animals (n = 28). Numbers within each cell represent Spearman’s correlation coefficients (ρ). Cell colors indicate the strength and direction of the correlations, with red representing positive correlations and blue representing negative correlations. All displayed correlations were statistically significant (*p* < 0.001, two-tailed).

To further examine the relationships among the principal mechanistic variables, Spearman’s rank correlation analysis was performed using pooled data from all experimental animals (n = 28). Hepatic SIRT1 levels were significantly and negatively correlated with NF-κB (ρ = −0.794, *p* < 0.001), HMGB1 (ρ = −0.677, *p* < 0.001), and hepatic ^99m^Tc-pyrophosphate (^99m^Tc-PYP) uptake (ρ = −0.788, *p* < 0.001). In contrast, hepatic ^99m^Tc-PYP uptake showed significant positive correlations with HMGB1 (ρ = 0.845, *p* < 0.001) and NF-κB (ρ = 0.818, *p* < 0.001), while HMGB1 was also positively correlated with NF-κB (ρ = 0.776, *p* < 0.001). Overall, these findings demonstrate strong associations among reduced SIRT1 expression, enhanced inflammatory signaling, and increased hepatic ^99m^Tc-PYP uptake. However, these correlations indicate statistical associations rather than causal relationships (Figure 5).

## 3. Discussion

The present findings indicate that acute doxorubicin exposure produced a consistent pattern of hepatic injury characterized by increased injury-associated enzyme activities in liver tissue, higher circulating HMGB1, enhanced oxidative and nitrosative stress, a pro-inflammatory cytokine profile, lower hepatic SIRT1 levels, and increased hepatic ^99m^Tc-pyrophosphate uptake. Exenatide attenuated each of these abnormalities, although none returned completely to control levels. The overall pattern, therefore, indicates attenuation of acute doxorubicin-induced hepatic injury rather than complete reversal of toxicity [2,3].

The oxidative stress profile observed after doxorubicin exposure was biologically coherent and consistent with acute hepatic oxidative injury. Doxorubicin increased MDA, TOS, and nitric oxide while depleting GSH and TAS, indicating a marked shift toward an oxidizing intracellular environment. This pattern is consistent with current concepts of doxorubicin hepatotoxicity, in which redox cycling, mitochondrial electron transport disruption, iron-dependent free radical formation, and membrane lipid peroxidation converge to promote hepatocellular injury [2,3]. Exenatide partially corrected these abnormalities, consistent with previous studies showing that GLP-1 receptor agonists reduce oxidative burden and improve antioxidant defenses, partly through mechanisms associated with Nrf2 signaling and mitochondrial stress adaptation [13,20]. Nevertheless, mitochondrial respiration, Nrf2 activation, and lipid peroxidation-related adducts were not directly evaluated in the present study; therefore, these mechanisms should be regarded as biologically plausible rather than experimentally confirmed.

In addition to oxidative stress, our findings support a contribution of nitrosative stress to doxorubicin-induced liver injury. The increased NO levels observed in the DOX group are consistent with enhanced nitric oxide production, potentially associated with NF-κB–dependent inflammatory signaling, a mechanism widely implicated in inflammatory tissue injury [21]. Excess NO can react with superoxide to form peroxynitrite, a highly reactive species that promotes lipid peroxidation, protein nitration, mitochondrial dysfunction, and DNA damage [3,21]. Exenatide reduced NO levels, consistent with attenuation of inflammatory activation. However, because iNOS expression and nitrotyrosine formation were not evaluated, the present findings should not be interpreted as direct evidence of inhibition of the NF-κB/iNOS pathway. Previous studies have nevertheless shown that exenatide can reduce iNOS activity and inflammatory mediator production in experimental inflammatory models [13,22].

The SIRT1, HMGB1, and NF-κB findings merit careful mechanistic interpretation. SIRT1 is a broad stress-response regulator in the liver and has been linked to suppression of inflammatory transcription, maintenance of mitochondrial homeostasis, antioxidant defense, and resistance to toxic injury [4,8]. In the present study, doxorubicin reduced hepatic SIRT1 while increasing HMGB1 and NF-κB, whereas exenatide shifted these markers in the opposite direction. This pattern is consistent with an association between higher hepatic SIRT1 levels and reduced inflammatory activation, but it does not establish that changes in SIRT1 were the primary driver of the downstream responses. No SIRT1 inhibitor or activator comparator, genetic manipulation, or acetylation-specific analysis was performed. Accordingly, the most appropriate interpretation is that changes in hepatic SIRT1 were associated with the observed biochemical improvements rather than representing direct evidence of a causal SIRT1-dependent mechanism.

Interpretation of the proposed SIRT1–HMGB1–NF-κB axis also requires caution. HMGB1 functions both as a marker of cellular injury and as an active amplifier of sterile inflammation through pattern-recognition pathways such as TLR4 and RAGE, leading to downstream NF-κB activation [7,9]. SIRT1 has been linked to HMGB1 regulation in several experimental settings, providing a biologically plausible connection between redox homeostasis and inflammatory amplification [9,16]. However, lower HMGB1 levels after exenatide treatment may reflect several non-mutually exclusive mechanisms, including attenuation of primary hepatocellular injury, altered HMGB1 trafficking, reduced inflammatory amplification, or broader improvement in redox homeostasis. Previous studies showing that exenatide can modulate SIRT1-related pathways in experimental liver disease further support the biological plausibility of this interpretation [15]. Nevertheless, the present findings remain associative and should not be interpreted as direct mechanistic evidence that exenatide acts through a single verified SIRT1–HMGB1–NF-κB signaling cascade.

Exenatide most likely acted through multiple interconnected mechanisms rather than a single verified signaling axis. Experimental studies have shown that GLP-1 receptor agonists modulate AMPK-related signaling, mitophagy, inflammasome activity, oxidative stress responses, and inflammatory gene expression in experimental liver injury models [4,12,23]. The present findings are compatible with this broader mechanistic framework, as exenatide improved oxidant–antioxidant balance, reduced nitric oxide and pro-inflammatory mediators, and increased IL-10 together with SIRT1. Some of these effects may reflect direct hepatic signaling, whereas others may result from systemic metabolic effects or modulation of immune cell function. However, the present study cannot distinguish among these possibilities because hepatocyte-specific signaling, Kupffer cell responses, macrophage polarization, and inflammasome activation were not directly evaluated.

The inflammatory cytokine profile further supports the presence of a persistent inflammatory response following doxorubicin exposure. Higher NF-κB, TNF-α, and IL-6 levels, together with lower IL-10, indicate a hepatic microenvironment shifted toward sustained inflammatory activation rather than balanced resolution [7,9]. Exenatide partially reversed each of these abnormalities, suggesting a less injurious inflammatory milieu rather than complete immunologic normalization. The partial recovery of IL-10 is particularly relevant because it may reflect restoration of anti-inflammatory counter-regulatory mechanisms even when tissue injury has not been fully resolved. Nevertheless, without immune-cell phenotyping, it remains uncertain whether these changes reflect altered hepatocyte signaling, macrophage polarization, non-parenchymal cell responses, or a combination of these mechanisms.

Evaluation of hepatic ^99m^Tc-pyrophosphate uptake is a novel aspect of the present study, but the findings should be interpreted as exploratory. ^99m^Tc-pyrophosphate is a necrosis-avid radiotracer that localizes within injured tissue microenvironments, primarily due to calcium overload, membrane disruption, binding to denatured macromolecules, and microcalcific change, rather than hepatocyte-specific functional transport [17,18]. Historical nuclear medicine studies also described hepatic uptake in severe hepatocellular necrosis, supporting the biological plausibility of the present observations [17,19]. In the present study, hepatic ^99m^Tc-pyrophosphate uptake increased following doxorubicin administration and decreased after exenatide treatment in parallel with the biochemical markers of hepatic injury. The concordance between scintigraphic and biochemical findings suggests that hepatic ^99m^Tc-pyrophosphate uptake may reflect the overall burden of acute tissue injury in this experimental model. However, the tracer is not hepatocyte-specific, contemporary liver imaging has evolved beyond this application, and the present findings were not validated against histopathology or established functional hepatic imaging methods. Accordingly, hepatic ^99m^Tc-pyrophosphate uptake should currently be regarded as an exploratory imaging correlate of acute tissue injury rather than an established biomarker of hepatic injury.

Correlation analysis demonstrated significant inverse associations between hepatic SIRT1 and both serum HMGB1 and hepatic NF-κB, whereas hepatic ^99m^Tc-pyrophosphate (^99m^Tc-PYP) uptake was positively correlated with serum HMGB1 and hepatic NF-κB. These associations support the internal biological consistency of the measured variables and are compatible with the proposed role of SIRT1-, HMGB1-, and NF-κB-related pathways in doxorubicin-induced liver injury. However, because the analyses were performed using pooled data from different experimental groups, the observed correlations should be interpreted as statistical associations rather than evidence of direct mechanistic interactions or causality and may partly reflect treatment-group differences.

Whether attenuation of hepatic injury might influence the antitumor efficacy of doxorubicin is an important translational consideration. The present study cannot address this question because it was performed in healthy rats and did not include tumor-bearing models or oncologic endpoints. Although a large retrospective study reported that GLP-1 receptor agonist use was associated with a lower incidence of several obesity-associated cancers in patients with type 2 diabetes [8], these observational findings cannot be extrapolated to the case of exenatide co-administration during doxorubicin chemotherapy. Likewise, they do not establish that exenatide has no effect on tumor biology, drug distribution, or the antitumor efficacy of doxorubicin. Therefore, studies using tumor-bearing models are required before any conclusions regarding the oncologic safety or therapeutic implications of this combination can be drawn.

### Study Limitations

This study has several limitations. First, histopathological and immunohistochemical evaluation of liver tissue was not performed; therefore, the biochemical and scintigraphic findings could not be validated against structural alterations in the tissue. Second, liver function indices, including serum albumin, bilirubin, ammonia, blood urea nitrogen, and coagulation parameters, were not assessed; consequently, the findings reflect attenuation of acute hepatic injury rather than recovery of global hepatic function. Third, the study employed a small sample size, a short-duration acute model, and a preventive pretreatment design without dose–response evaluation. In addition, comprehensive analytical validation parameters for the HPLC-based MDA assay were not prospectively documented. Finally, only male rats were studied, and no tumor-bearing model or mechanistic intervention using SIRT1 inhibitors, activators, or genetic approaches was included. Future studies incorporating histopathology, immunohistochemistry, liver function assessment, mechanistic validation, longer-term treatment protocols, and tumor-bearing models are required to define the translational potential of exenatide in doxorubicin-induced liver injury.

## 4. Materials and Methods

### 4.1. Animal

Twenty-eight male Wistar albino rats weighing 230–250 g were used in this study (n = 7 per group). Animals were housed under standard laboratory conditions (22–25 °C, 12 h light/dark cycle) with free access to food and water and were acclimatized for one week before the experiment. Experimental procedures were conducted in accordance with the ARRIVE 2.0 framework for reporting animal studies and were approved by the Tokat Gaziosmanpaşa University Local Animal Ethics Committee (Approval No: 51878863-11; Approval Date: 15 January 2018).

### 4.2. Dose and Treatment Rationale

The exenatide dose was selected based on previous experimental studies demonstrating hepatoprotective and anti-inflammatory effects of GLP-1 receptor agonists in rodents [13,14,24]. In contrast, the doxorubicin regimen was selected because it has been widely used to induce reproducible acute hepatic injury within a short experimental period [10,25].

The five-day pretreatment phase was intentional and was designed to evaluate whether prior GLP-1 receptor activation could prime antioxidant and anti-inflammatory defense pathways before acute doxorubicin challenge. Accordingly, the present design tests preventive or attenuating efficacy rather than therapeutic rescue after injury has already been established. The total seven-day treatment period was selected to capture acute doxorubicin-induced biochemical injury while maintaining comparability with short-duration experimental toxicity protocols.

### 4.3. Experimental Groups and Treatment Protocols

Animals were acclimatized for one week before the study and monitored daily throughout the experimental period in accordance with institutional animal welfare practices. Animals were assigned to four groups: control, exenatide, doxorubicin, and exenatide + doxorubicin (n = 7/group). Group allocation was performed before treatment initiation using a simple random assignment procedure. Outcome assessment was performed using coded samples and coded scintigraphic images to reduce observer bias during biochemical and imaging analyses.

Animals were randomly allocated into four experimental groups:

Group I (Control):

Rats in the control group received no active pharmacological intervention and were administered equivalent volumes of intraperitoneal saline throughout the experimental period.

Group II (Exenatide):

Animals received exenatide at a dose of 10 µg/kg/day via intraperitoneal injection, administered once daily at 09:00 for a total duration of seven consecutive days.

Group III (Doxorubicin):

Doxorubicin was administered intraperitoneally on days 5, 6, and 7 of the experimental protocol, reaching a cumulative dose of 18 mg/kg.

**Figure 6:**
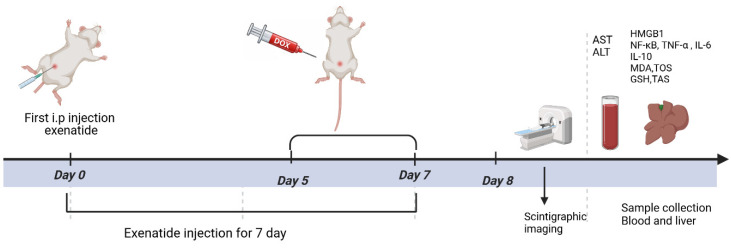
Experimental timeline. Exenatide (10 µg/kg/day, intraperitoneally) was administered once daily for seven consecutive days. Doxorubicin was administered intraperitoneally on days 5, 6, and 7 at a cumulative dose of 18 mg/kg. On day 8, ^99m^Tc-pyrophosphate scintigraphy was performed, followed by blood collection and liver tissue harvesting.

Group IV (Exenatide + Doxorubicin):

Rats in this group were treated with exenatide (10 µg/kg/day, i.p.) once daily at 09:00 for seven consecutive days. Additionally, doxorubicin was administered intraperitoneally on days 5, 6, and 7, in accordance with the same dosing regimen applied in the doxorubicin-only group (cumulative dose: 18 mg/kg) [26] (Figure 6).

### 4.4. Timing of Imaging and Tissue Sampling

On day 8, approximately 24 h after the last drug administration, scintigraphic imaging was performed. After completion of imaging, animals were euthanized under the approved experimental protocol, blood was collected from the abdominal aorta, and liver tissue was harvested immediately for biochemical analysis. Samples were processed on the day of collection or stored under appropriate cold-chain conditions until analysis.

### 4.5. Scintigraphic Imaging

Following completion of the experimental protocol, scintigraphic imaging was performed. ^99m^Tc-pyrophosphate (^99m^Tc-PYP; TechneScan PYP, Mallinckrodt, Québec City, QC, Canada) was prepared by diluting 1 mCi of the radiotracer in 5 mL of isotonic saline. From this preparation, 0.1 mL was administered intraperitoneally to each animal. Static scintigraphic images were acquired one hour after tracer injection using a gamma camera system (Siemens Symbia, Erlangen, Germany).

For quantitative analysis, a manually placed circular region of interest (ROI) was positioned over the anatomical projection of the liver on each static image, using the anatomical positions of the kidneys and urinary bladder as reference landmarks to ensure consistent ROI placement. Care was taken to avoid inclusion of adjacent non-hepatic tracer activity during ROI placement. ROI measurements were performed independently by two observers who were blinded to group allocation throughout image analysis. Each observer independently placed the ROI seven times for each animal, and the mean hepatic count value from these repeated measurements was used for statistical analysis to minimize measurement variability. Group identities were disclosed only after completion of all image analyses. Because ^99m^Tc-pyrophosphate is not a hepatocyte-specific tracer, hepatic uptake values were interpreted as relative indicators of hepatic tissue injury rather than validated measures of hepatic function.

### 4.6. Biochemical Analysis

#### 4.6.1. Biochemical Analysis of Liver Enzymes

Blood samples (~5 mL) were collected from the abdominal aorta into serum-separator tubes and allowed to clot for 30 min. Samples were then centrifuged at 1500× *g* for 10 min, and the obtained serum was used for subsequent analyses. Serum HMGB1 was measured using commercially available rat-specific ELISA kits (IBL International GmbH, Hamburg, Germany) according to the manufacturer’s protocols. 

#### 4.6.2. Biochemical Analysis of Liver Tissue

Liver samples were immediately rinsed with ice-cold physiological saline, weighed, and homogenized in ice-cold phosphate-buffered saline (PBS; pH 7.4) using stainless steel beads. The homogenates were centrifuged at 2500 rpm for 20 min at 4 °C, and the resulting supernatants were collected for subsequent biochemical analyses. Total protein concentration was determined for each homogenate, and all tissue-derived biochemical parameters were normalized to protein content and expressed per milligram of protein (mg protein).

Intrahepatic ALT and AST activities were determined in liver homogenates using commercially available assay kits and expressed as U/mg protein. These measurements were used to reflect injury-associated intrahepatic enzymatic alterations and should not be interpreted as conventional serum liver function tests.

Tumor necrosis factor-α (TNF-α), interleukin-6 (IL-6), and nitric oxide (NO) levels were determined using commercially available sandwich ELISA kits (Bioassay Technology Laboratory, Shanghai, China; Cat. No: E0764Ra, E0135Ra, and E0703Ra, respectively), in accordance with the manufacturer’s protocols. Nuclear factor kappa B (NF-κB p65) levels were measured using a rat-specific ELISA kit (MyBioSource, USA; Cat. No: MBS2508000). (MyBioSource, San Diego, CA, USA) Sirtuin-1 (SIRT-1) concentrations were quantified using a rat ELISA kit (Elabscience, Houston, TX, USA; Cat. No: E-EL-R1102). Total antioxidant status (TAS) and total oxidant status (TOS) were assessed using automated colorimetric assay kits (Rel Assay Diagnostics, Gaziantep, Turkey). Tissue reduced glutathione (GSH) levels were determined using a commercially available colorimetric assay kit (Invitrogen/Thermo Fisher Scientific, Cat. No. EEA020; (Invitrogen, Thermo Fisher Scientific, Waltham, MA, USA) in accordance with the manufacturer’s protocol. IL-10 was measured using commercially available rat-specific ELISA kits according to the manufacturers’ protocols.

#### 4.6.3. Malondialdehyde

Malondialdehyde was quantified using an HPLC-assisted thiobarbituric acid reactive substances method based on previously published and analytically validated protocols [14,27]. Samples underwent acid hydrolysis and heating to liberate bound malondialdehyde, followed by derivatization with thiobarbituric acid. Proteins were precipitated with methanol, samples were centrifuged, and the supernatant was injected onto a C18 reversed-phase column. The malondialdehyde–TBA_2_ adduct was detected at 532 nm and quantified using tetraethoxypropane external calibration. Duplicate analyses and routine quality-control procedures were performed throughout sample processing. However, although the analytical method itself has been previously validated, complete laboratory-specific validation parameters (e.g., linearity range, recovery, and formal limits of detection and quantification) from the original experimental records were not prospectively archived and therefore cannot be reported in detail. This should be considered when interpreting the MDA data.

### 4.7. Statistical Analysis

Statistical analyses were performed using SPSS version 19 and GraphPad Prism version 9. Data distribution was assessed using the Shapiro–Wilk test, confirming normality across all groups. Accordingly, comparisons were conducted using one-way ANOVA followed by Tukey’s post hoc test. Results are presented as mean ± SEM, and *p* < 0.05 was considered statistically significant.

#### Sample Size Calculation

The sample size was determined with reference to previous experimental studies of doxorubicin-induced toxicity using comparable experimental protocols, cumulative doxorubicin doses, and group sizes [28,29]. To further support the selected sample size, an a priori power analysis was performed using G*Power software (version 3.1). Assuming a one-way ANOVA design, a two-sided α level of 0.05, a statistical power of 80%, a large effect size (Cohen’s f = 0.40), the analysis indicated that at least five animals per group were required. Therefore, seven animals were included in each group (total n = 28) to provide adequate statistical power while accounting for potential biological variability.

## 5. Conclusions

Exenatide attenuated doxorubicin-induced acute hepatic injury in this rat model and was associated with lower oxidative and nitrosative stress, reduced inflammatory activation, higher hepatic SIRT1 levels, lower serum HMGB1 levels, and reduced hepatic ^99m^Tc-pyrophosphate uptake. The overall pattern of findings is consistent with involvement of SIRT1–HMGB1/NF-κB-related signaling; however, direct mechanistic causality was not established, and multiple interconnected pathways are likely to have contributed to the observed effects. Hepatic ^99m^Tc-pyrophosphate uptake behaved as an exploratory imaging correlate of tissue injury and requires further validation against histopathology and established hepatic imaging methods. Overall, these findings support further investigation of exenatide as a potential strategy for attenuating doxorubicin-induced acute hepatic injury.

## Figures and Tables

**Table 1:** Biochemical and scintigraphic markers of hepatic injury in the experimental groups (Mean ± SEM).

Parameter	Control	Exenatide	DOX	Exenatide + DOX
Hepatic ALT activity (U/mg protein)	0.62 ± 0.04	0.66 ± 0.04	2.61 ± 0.26 ***	1.37 ± 0.14 **^,###^
Hepatic AST activity (U/mg protein)	1.82 ± 0.15	1.86 ± 0.16	8.78 ± 0.34 ***	5.28 ± 0.74 ***^,###^
HMGB1	0.96 ± 0.16	0.99 ± 0.09	3.46 ± 0.34 ***	1.79 ± 0.14 *^,###^
Scintigraphy	42,874 ± 2956	39,929 ± 2657	243,609 ± 14,897 ***	125,081 ± 12,404 ***^,###^

Data are presented as mean ± SEM (n = 7/group). Statistical comparisons among groups were performed using one-way ANOVA followed by Tukey’s post hoc test. * *p* < 0.05, ** *p* < 0.01, *** *p* < 0.001 vs. control; ^###^ *p* < 0.001 vs. DOX.

**Table 2:** Hepatic inflammatory pathway markers in the experimental groups (Mean ± SEM).

Parameter	Control	Exenatide	DOX	Exenatide + DOX
NF-κB	0.372 ± 0.038	0.368 ± 0.030	1.125 ± 0.093 ***	0.671 ± 0.053 ***^,##^
TNF-α	25.44 ± 4.85	26.32 ± 3.31	133.73 ± 15.02 ***	68.73 ± 6.26 **^,###^
IL-6	4.29 ± 0.54	3.93 ± 0.61	15.58 ± 1.63 ***	9.17 ± 0.66 **^,###^
IL-10	12.90 ± 1.12	13.06 ± 1.18	4.53 ± 0.39 ***	9.74 ± 0.67 **^,###^
SIRT1	3.42 ± 0.21	3.66 ± 0.19	1.20 ± 0.11 ***	2.30 ± 0.25 ^##^

Data are presented as mean ± SEM (n = 7 per group). Statistical comparisons among groups were performed using one-way ANOVA followed by Tukey’s post hoc test. ** *p* < 0.01, *** *p* < 0.001 vs. control; ^##^ *p* < 0.01, ^###^ *p* < 0.001 vs. DOX.

**Table 3:** Hepatic oxidative and nitrosative stress markers in the experimental groups.

Parameter	Control	Exenatide	DOX	Exenatide + DOX
MDA	1.59 ± 0.19	1.44 ± 0.17	5.69 ± 0.45 ***	3.13 ± 0.30 **^,###^
GSH	25.93 ± 1.56	28.36 ± 1.05	8.85 ± 1.03 ***	19.03 ± 1.47 **^,###^
TAS	1.26 ± 0.10	1.38 ± 0.09	0.49 ± 0.05 ***	0.92 ± 0.06 *^,##^
TOS	6.86 ± 0.49	6.30 ± 0.32	16.48 ± 0.52 ***	10.34 ± 0.66 ***^,###^
NO	65.88 ± 3.99	62.81 ± 3.07	148.01 ± 14.47 ***	87.80 ± 5.44 ^###^

Data are presented as mean ± SEM (n = 7/group). Statistical comparisons among groups were performed using one-way ANOVA followed by Tukey’s post hoc test. * *p* < 0.05, ** *p* < 0.01, *** *p* < 0.001 vs. control; ^##^ *p* < 0.01, ^###^ *p* < 0.001 vs. DOX.

## Data Availability

The data that support the findings of this study are not publicly available due to ethical reasons but are available from the corresponding author upon request.

## References

[1] Bray F., Laversanne M., Sung H., Ferlay J., Siegel R.L., Soerjomataram I., Jemal A. (2024). Global cancer statistics 2022: GLOBOCAN estimates of incidence and mortality worldwide for 36 cancers in 185 countries. CA Cancer J. Clin..

[2] Radeva L., Yoncheva K. (2025). Doxorubicin toxicity and recent approaches to alleviating its adverse effects with focus on oxidative stress. Molecules.

[3] Prasanna P.L., Renu K., Gopalakrishnan A.V. (2020). New molecular and biochemical insights of doxorubicin-induced hepatotoxicity. Life Sci..

[4] El-Dessouki A.M., Yousef E.H., Raslan N.A., Alwakeel A.I., Ibrahim S., Alzokaky A.A. (2025). Febuxostat protects from Doxorubicin induced hepatotoxicity in rats via regulation of NF-κB p65/NLRP3 inflammasome and SIRT-1/AMPK pathways. Naunyn-Schmiedebergs Arch. Pharmacol..

[5] Laftah A.H., Alhelfi N., Al Salait S.K., Altemimi A.B., Tabandeh M.R., Tsakali E., Van Impe J.F., El-Maksoud A.A.A., Abedelmaksoud T.G. (2025). Mitigation of doxorubicin-induced liver toxicity in mice breast cancer model by green tea and Moringa oleifera combination: Targeting apoptosis, inflammation, and oxidative stress. J. Funct. Foods.

[6] Saleh D.O., Mahmoud S.S., Hassan A., Sanad E.F. (2022). Doxorubicin-induced hepatic toxicity in rats: Mechanistic protective role of Omega-3 fatty acids through Nrf2/HO-1 activation and PI3K/Akt/GSK-3β axis modulation. Saudi J. Biol. Sci..

[7] Ni Y.-A., Chen H., Nie H., Zheng B., Gong Q. (2021). HMGB1: An overview of its roles in the pathogenesis of liver disease. J. Leukoc. Biol..

[8] Wang M., Zhao J., Chen J., Long T., Xu M., Luo T., Che Q., He Y., Xu D. (2024). The role of sirtuin1 in liver injury: Molecular mechanisms and novel therapeutic target. PeerJ.

[9] Wei L., Zhang W., Li Y., Zhai J. (2022). The SIRT1-HMGB1 axis: Therapeutic potential to ameliorate inflammatory responses and tumor occurrence. Front. Cell Dev. Biol..

[10] Song S., Chu L., Liang H., Chen J., Liang J., Huang Z., Zhang B., Chen X. (2019). Protective effects of dioscin against doxorubicin-induced hepatotoxicity via regulation of Sirt1/FOXO1/NF-κb signal. Front. Pharmacol..

[11] Yang Y., Liu Y., Wang Y., Chao Y., Zhang J., Jia Y., Tie J., Hu D. (2022). Regulation of SIRT1 and its roles in inflammation. Front. Immunol..

[12] Shao N., Yu X.Y., Ma X.F., Lin W.J., Hao M., Kuang H.Y. (2018). Exenatide Delays the Progression of Nonalcoholic Fatty Liver Disease in C57BL/6 Mice, Which May Involve Inhibition of the NLRP3 Inflammasome through the Mitophagy Pathway. Gastroenterol. Res. Pract..

[13] Saad Z.A., Khodeer D.M., Zaitone S.A., Ahmed A.A., Moustafa Y.M. (2020). Exenatide ameliorates experimental non-alcoholic fatty liver in rats via suppression of toll-like receptor 4/NFκB signaling: Comparison to metformin. Life Sci..

[14] Gupta N.A., Kolachala V.L., Jiang R., Abramowsky C., Romero R., Fifadara N., Anania F., Knechtle S., Kirk A. (2012). The glucagon-like peptide-1 receptor agonist Exendin 4 has a protective role in ischemic injury of lean and steatotic liver by inhibiting cell death and stimulating lipolysis. Am. J. Pathol..

[15] Xu F., Li Z., Zheng X., Liu H., Liang H., Xu H., Chen Z., Zeng K., Weng J. (2014). SIRT1 mediates the effect of GLP-1 receptor agonist exenatide on ameliorating hepatic steatosis. Diabetes.

[16] Hwang J.S., Choi H.S., Ham S.A., Yoo T., Lee W.J., Paek K.S., Seo H.G. (2015). Deacetylation-mediated interaction of SIRT1-HMGB1 improves survival in a mouse model of endotoxemia. Sci. Rep..

[17] Hansen S., Stadalnik R.C. (1982). Liver uptake of ^99m^Tc-pyrophosphate. Semin. Nucl. Med..

[18] Buja L.M., Tofe A.J., Kulkarni P.V., Mukherjee A., Parkey R.W., Francis M.D., Bonte F.J., Willerson J.T. (1977). Sites and mechanisms of localization of technetium-99m phosphorus radiopharmaceuticals in acute myocardial infarcts and other tissues. J. Clin. Investg..

[19] Lyons K.P., Kuperus J., Green H.W. (1977). Localization of Tc-99m-pyrophosphate in the liver due to massive liver necrosis: Case report. J. Nucl. Med..

[20] Oh Y.S., Jun H.S. (2017). Effects of glucagon-like peptide-1 on oxidative stress and Nrf2 signaling. Int. J. Mol. Sci..

[21] Mukhopadhyay P., Rajesh M., Bátkai S., Kashiwaya Y., Haskό G., Liaudet L., Szabó C., Pacher P. (2009). Role of superoxide, nitric oxide, and peroxynitrite in doxorubicin-induced cell death in vivo and in vitro. Am. J. Physiol.-Heart Circ. Physiol..

[22] Bułdak Ł., Łabuzek K., Bułdak R.J., Machnik G., Bołdys A., Okopień B. (2015). Exenatide (a GLP-1 agonist) improves the antioxidative potential of in vitro cultured human monocytes/macrophages. Naunyn-Schmiedebergs Arch. Pharmacol..

[23] Arab H.H., Eid A.H., Alsufyani S.E., Ashour A.M., Alnefaie A.M., Alsharif N.M., Alshehri A.M., Almalawi A.A., Alsowat A.A., Abd El Aal H.A. (2024). Activation of AMPK/mTOR-Driven Autophagy and Suppression of the HMGB1/TLR4 Pathway with Pentoxifylline Attenuates Doxorubicin-Induced Hepatic Injury in Rats. Pharmaceuticals.

[24] Ding X., Saxena N.K., Lin S., Gupta N., Anania F.A. (2006). Exendin-4, a glucagon-like protein-1 (GLP-1) receptor agonist, reverses hepatic steatosis in ob/ob mice. Hepatology.

[25] Alshabanah O.A., Hafez M.M., Al-Harbi M.M., Hassan Z.K., Al Rejaie S.S., Asiri Y.A., Sayed-Ahmed M.M. (2010). Doxorubicin toxicity can be ameliorated during antioxidant L-carnitine supplementation. Oxidative Med. Cell. Longev..

[26] Koroglu R., Koroglu M., Aygun H. (2024). Electrocardiographic, biochemical, and scintigraphic evidence for the cardioprotective effect of paricalcitol and vitamin D3 on doxorubicin-induced acute cardiotoxicity in rats. Bratisl. Med. J. Bratisl. Lek. Listy.

[27] Damodar G., Smitha T., Gopinath S., Vijayakumar S., Rao Y.A. (2014). An evaluation of hepatotoxicity in breast cancer patients receiving injection Doxorubicin. Ann. Med. Health Sci. Res..

[28] Podyacheva E.Y., Kushnareva E.A., Karpov A.A., Toropova Y.G. (2021). Analysis of Models of Doxorubicin-Induced Cardiomyopathy in Rats and Mice. A Modern View from the Perspective of the Pathophysiologist and the Clinician. Front. Pharmacol..

[29] Gül S.S., Aygün H. (2018). Cardioprotective effect of vitamin D and melatonin on doxorubicin-induced cardiotoxicity in rat model: An electrocardiographic, scintigraphic and biochemical study. Eur. Res. J..

